# An Alternative Statistical Characterization of TWDP Fading Model

**DOI:** 10.3390/s21227513

**Published:** 2021-11-12

**Authors:** Almir Maric, Enio Kaljic, Pamela Njemcevic

**Affiliations:** Department of Telecommunications, Faculty of Electrical Engineering, University of Sarajevo, Zmaja od Bosne b.b., Kampus Univerziteta, 71000 Sarajevo, Bosnia and Herzegovina; enio.kaljic@etf.unsa.ba (E.K.); pamela.njemcevic@etf.unsa.ba (P.N.)

**Keywords:** TWDP fading channel, MGF, M-ary PSK, ASEP

## Abstract

Two-wave with diffuse power (TWDP) is one of the most promising models for the description of small-scale fading effects in 5G networks, which employs mmWave band, and in wireless sensor networks deployed in different cavity environments. However, its current statistical characterization has several fundamental issues. Primarily, conventional TWDP parameterization is not in accordance with the model’s underlying physical mechanisms. In addition, available TWDP expressions for PDF, CDF, and MGF are given either in integral or approximate forms, or as mathematically untractable closed-form expressions. Consequently, the existing TWDP statistical characterization does not allow accurate evaluation of system performance in all fading conditions for most modulation and diversity techniques. In this regard, physically justified TWDP parameterization is proposed and used for further calculations. Additionally, exact infinite-series PDF and CDF are introduced. Based on these expressions, the exact MGF of the SNR is derived in a form suitable for mathematical manipulations. The applicability of the proposed MGF for derivation of the exact average symbol error probability (ASEP) is demonstrated with the example of M-ary PSK modulation. The derived M-ary PSK ASEP expression is further simplified for large SNR values in order to obtain a closed-form asymptotic ASEP, which is shown to be applicable for SNR > 20 dB. All proposed expressions are verified by Monte Carlo simulation in a variety of TWDP fading conditions.

## 1. Introduction

Due to the tremendous growth of Internet data traffic, bandwidth requirements have become especially pronounced. To cope with these requirements, the fifth generation (5G) mobile network is emerging as the latest wireless communication standard. At the heart of this technology lies the use of the millimeter wave (mmWave) frequency band.

However, a signal propagating in a mmWave band exhibits unique propagation properties, making traditional small-scale fading models inadequate and thus demanding more generalized models. To address this issue, Durgin et al. [1] proposed the two-wave with diffuse power (TWDP) model, which assumes that the complex envelopes consist of two strong specular components and many weak diffuse components. As such, it encompasses Rayleigh, Rician, and two-ray fading models as its special cases [1], simultaneously enabling modeling of both worse-than-Rayleigh and Rician-like fading conditions.

In the last twenty years, the TWDP model has been extensively studied theoretically [2,3,4,5,6,7,8,9,10,11,12,13,14,15,16,17,18,19,20]. Additionally, its existence is supported by practical evidence both in mmWave 5G communication networks equipped with directional antennas or arrays [21] and in wireless sensor networks deployed in cavity environments [22]. Namely, the applicability of TWDP for modeling mmWave outdoor radio propagation channel is verified by ray-tracing simulation for modeling train-to-infrastructure wireless communications [23] and for modeling vehicle-to-vehicle communication in urban environments at 60 GHz [24]. Two indoor mmWave measurement campaigns performed in [25] revealed that the TWDP model is also adequate for modeling indoor mmWave communication. Therewith, the TWDP fading model is also the best choice for modeling near-body mmWave channels, both in front and back regions [26]. In addition, TWDP fading conditions are also met in static sensor networks with their nodes placed within cavity environments, such as aircrafts and buses [22] and abroad a large transport helicopter [27].

However, despite the fact that there are more than 5000 TWDP related results on Google, to the best of the authors’ knowledge, there are at least two factors that motivate further studies of TWDP fading and its performance:

1 Existing TWDP parameterization is not in accordance with the model’s underlying physical mechanisms,
2 Analytical forms of the existing expressions for PDF and MGF disallow accurate evaluation of the effects of TWDP fading on system performance.

To describe TWDP fading, Durgin et al. [1] proposed two parameters, K≥0 and 0≤Δ≤1, which reflect the relationship between specular and diffuse components, and between the specular components themselves, respectively. However, from the definition of parameter Δ, it is obvious that it introduces a nonlinear relationship between magnitudes of specular components, which, according to the underlying physical mechanisms of TWDP, has to be linear. Consequently, for a significant range of values (0≤Δ≤0.5), the effect of Δ increments on the system performance metrics (e.g., ASEP and outage probability) is almost unobservable (in fact, from ([2], Figure 7) it can be seen that the corresponding curves almost overlap for the considered range of Δ). This is obviously counterintuitive considering the physical meaning attributed to the parameter Δ. It is thus essential to examine the TWDP parameterization problem in depth.

Regarding TWDP PDF expressions, they are primarily proposed in integral ([1], Equation (29)), ([1], Equation (32)), ([2], Equation (16)), and approximate ([1], Equation (17)) forms. Therefore, the exact evaluation of system performance metrics based on the existing integral expressions is not mathematically tractable, disabling direct observation of TWDP fading effects on system performance. Accordingly, the closed-form results of performance evaluation (e.g., error and outage probability, etc.) are mostly obtained in approximate forms [3,4,5,6,7,8,9,10,11,12,13,14], derived using the approximate PDF expression. However, it has been shown that analysis based on an approximate PDF expression is accurate only for a narrow range of *K* and Δ values [1,15], which can only be used for the description of limited fading conditions.

To overcome these limitations, Rao et al. [16] proposed an alternative approach to statistical characterization of TWDP fading based on the observation that the TWDP fading model can be expressed in terms of a conditional underlying Rician distribution. Thus, by invoking the observed similarities and the existing expressions of Rician fading, Rao et al. derived a novel form of TWDP MGF expression ([16], Equation (25)). Thereby, in contrast to previously derived approximate MGF expressions ([11], Equation (8)) ([5], Equation (12)), the one proposed in [16] is given as a simple closed-form solution. However, this form is also not suitable for mathematical manipulations, and consequently, for calculation of the exact ASEP expressions for most modulation and diversity schemes. Accordingly, most of the existing results regarding ASEP are provided in integral form, which may not always be convenient for practical purposes [17]. The exceptions are ASEP expressions for M-ary FKS and DBPSK modulations, derived in [2] and [16], respectively.

Recently, the exact infinite-series TWDP PDF expression was proposed ([17], Equation (6)) and used for derivation of the exact infinite-series PDF expression of the extended generalized fluctuating two-ray (FTR) fading with arbitrarily distributed phases of specular components ([18], Equation (22)). However, similarly to the exact MGF from [16], the exact FTR PDF expression is used mostly for derivation of the exact ABEPs of binary modulated signals [18,28,29], approximate ASEP for M-ary PSK modulation [19] and the exact expression for QAM ASEP [20] expressed in terms of bivariate Meijer’s G function, which is difficult to compute in common software packages (such as Matlab, Mathematica and Maple) since it is not a built-in function [20].

Accordingly, in order to accurately evaluate the effects of TWDP fading on error rate performances, it is of tremendous importance to provide mathematically tractable PDF and MGF expressions.

Considering the above, our contributions are as follows:

1 We proposed alternative TWDP parameterization, which is in accordance with the model’s underlying physical mechanisms.
2 We introduced the exact convergent infinite-series TWDP envelope PDF and CDF expressions (previously derived in [30,31]).
3 We derived the alternative exact form of SNR MGF based on the adopted CDF expression and proposed parameterization, which is shown to be suitable for mathematical manipulations.
4 Based on the obtained MGF, we derived M-ary PSK ASEP in exact infinite-series form, which is, to the best of our knowledge, the first such expression proposed to date.
5 We also derived asymptotic M-ary PSK ASEP as a simple closed-form expression, which tightly follows the exact one for the practical range of SNR values, i.e., for SNR > 20 dB.

The rest of the paper is structured as follows. In Section 2, the TWDP fading model is introduced and statistically described using alternative envelope PDF and CDF expressions, given in terms of newly proposed parameters. The alternative MGF of the SNR expression is derived in Section 3. In Section 4, the applicability of the proposed MGF for accurate performance analysis is demonstrated by deriving the exact and asymptotic M-ary PSK ASEP expressions, which are then verified by Monte Carlo simulation. The main conclusions are outlined in Section 5.

## 2. TWDP Fading Model

In the slow, frequency nonselective fading channel with TWDP statistic, the complex envelope r(t) is composed of two strong specular components: LOS $v_{1}\left(t\right)$ and reflected $v_{2}\left(t\right)$, and many low-power diffuse components treated as a random process n(t):

(1) $$\begin{array}{cc} r(t) & =v_{1}\left(t\right)+v_{2}\left(t\right)+n\left(t\right)\\ & =V_{1}\exp\left(j\Phi_{1}\right)+V_{2}\exp\left(j\Phi_{2}\right)+n\left(t\right) \end{array}$$

Specular components are assumed to have constant magnitudes $V_{1}$ and $V_{2}$ and uniformly distributed phases $\Phi_{1}$ and $\Phi_{2}$ in [0,2π), while diffuse components are treated as a complex zero-mean Gaussian random process n(t) with average power $2\sigma^{2}$. Consequently, the average power of a signal r(t) is equal to $\Omega =V_{1}^{2}+V_{2}^{2}+2\sigma^{2}$.

### 2.1. The Revision of Parameter Δ

Conventional parameterization of TWDP fading, originally proposed in [1], introduced two parameters:

(2) $$K≜\frac{\mathrm{average}\;\mathrm{specular}\;\mathrm{power}}{\mathrm{diffuse}\;\mathrm{power}}=\frac{V_{1}^{2}+V_{2}^{2}}{2\sigma^{2}}$$

(3) $$\Delta ≜\frac{\mathrm{peak}\;\mathrm{specular}\;\mathrm{power}}{\mathrm{average}\;\mathrm{specular}\;\mathrm{power}}-1=\frac{2V_{1}V_{2}}{V_{1}^{2}+V_{2}^{2}}$$

Parameter *K*, (0≤K<∞), such as in the Rician fading model, characterizes TWDP fading severity. Parameter Δ, (0≤Δ≤1) for $V_{1}\geq 0$, $V_{2}\geq 0$, and $V_{2}\leq V_{1}$, implicitly characterizes the relationship between the magnitudes of specular components. However, the physical justification of the relationship between $V_{1}$ and $V_{2}$, introduced by the definition of parameter Δ in (3), is questionable. Namely, according to [32] “*for 0<Δ<1 there is a nonlinear relation between the magnitude of the specular components $V_{1}$ and $V_{2}$, i.e., $V_{2}=V_{1}\left(1-\sqrt{1-\Delta^{2}}\right)/\Delta$. However, the physical facts suggest a different conclusion about the relation between $V_{1}$ and $V_{2}$. In particular, according to the model for TWDP fading, specular components are constant, and they are a consequence of specific propagation conditions. Since an electromagnetic wave is propagating in a linear medium, a natural choice to appropriately characterize the relation between magnitudes $V_{1}$ and $V_{2}$ is given by $\Gamma ≜V_{2}/V_{1}$, where $V_{2}\leq V_{1}$. Seemingly, parameters Δ and Γ are both motivated by physical arguments. However, they do not have the same level of physical intuition.*” Hence, since $v_{2}\left(t\right)$ is nothing but a reflection of an LOS component $v_{1}\left(t\right)$ and both are propagating over the linear medium, the relation between their magnitudes can be nothing but linear. Accordingly, in contrast to parameter Δ, suggested parameter Γ—defined as a reflection coefficient $\Gamma =V_{2}/V_{1}$—does not violate the natural relation between $V_{1}$ and $V_{2}$.

Based on the above, it is now necessary to investigate the impact of nonlinear Δ-based parameterization of TWDP statistics.

Accordingly, parameters *K* and Δ are written in terms of $V_{2}/V_{1}$, as:

(4) $$\begin{array}{c} K=\frac{V_{1}^{2}+V_{2}^{2}}{2\sigma^{2}}=\frac{V_{1}^{2}}{2\sigma^{2}}\left[1+{\left(\frac{V_{2}}{V_{1}}\right)}^{2}\right]=K_{Rice}\left(1+\Gamma^{2}\right) \end{array}$$

(5) $$\Delta =\frac{2\frac{V_{2}}{V_{1}}}{1+{\left(\frac{V_{2}}{V_{1}}\right)}^{2}}$$

where $\Gamma =V_{2}/V_{1}$ and $K_{Rice}=V_{1}^{2}/\left(2\sigma^{2}\right)$ represent the Rician parameter *K* of a dominant specular component (introduced in this analysis only in order to provide clearer observations). Based on the above, parameter *K* is also expressed in terms of Δ, as:

(6) $$\begin{array}{cc} K & =\frac{V_{1}^{2}+V_{2}^{2}}{2\sigma^{2}}=\frac{1}{2\sigma^{2}}\frac{2V_{1}V_{2}}{\Delta }\frac{V_{1}}{V_{1}}=\frac{V_{1}^{2}}{2\sigma^{2}}\frac{2}{\Delta }\frac{V_{2}}{V_{1}}=\\ & =K_{Rice}2\frac{1-\sqrt{1-\Delta^{2}}}{\Delta^{2}} \end{array}$$

**Figure 1:**
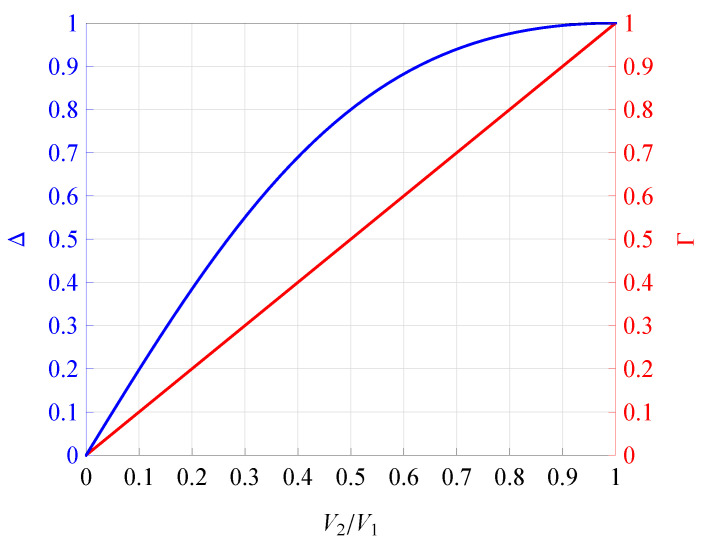
Dependence of Δ and Γ on $V_{2}/V_{1}$.

Figure 1 illustrates the functional dependence of parameter Δ versus $V_{2}/V_{1}$ (5). In the same figure, the linear dependence of Γ on $V_{2}/V_{1}$ is also illustrated as a benchmark. From Figure 1, it is evident that for $0<V_{2}/V_{1}<1$, Δ differs Γ not only in value, but also in terms of the character of their functional dependence on $V_{2}/V_{1}$. Consequently, when $V_{2}/V_{1}$ changes from 0.6 to 1, Δ changes only between 0.9 and 1. In general, for $0<V_{2}/V_{1}<1$, Δ is always greater than Γ.

**Figure 2:**
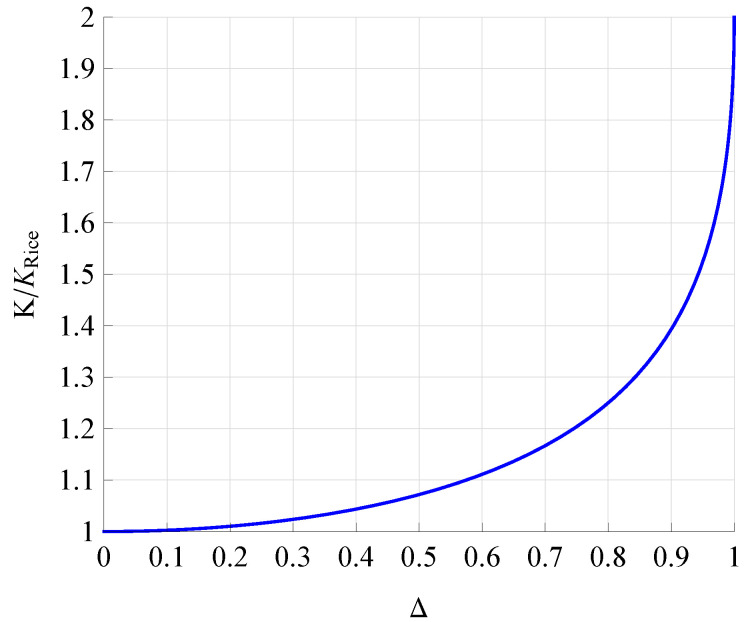
Dependence of $K/K_{Rice}$ on Δ.

**Figure 3:**
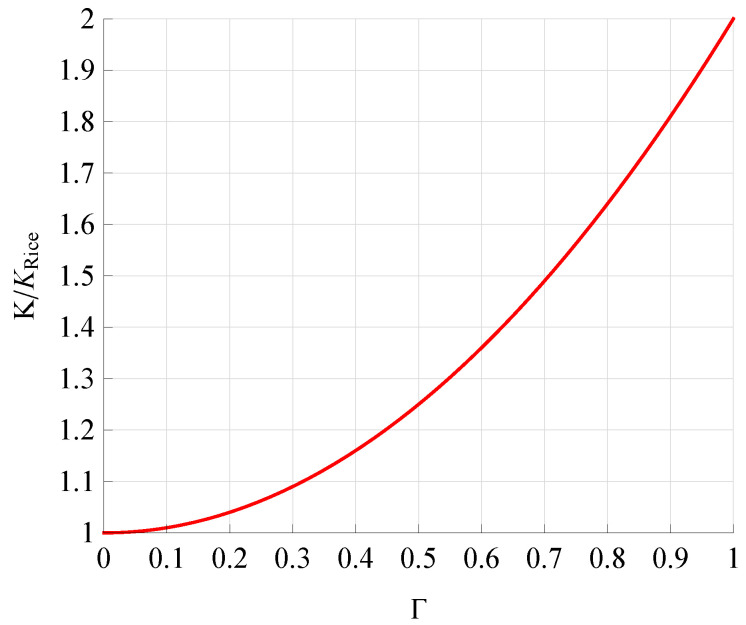
Dependence of $K/K_{Rice}$ on Γ.

Figure 2 and Figure 3 illustrate the dependencies of the normalized parameter *K* ($K/K_{Rice}$) on Δ (6) and Γ (4), which are clearly very different. For Δ≤0.8, *K* vs. Δ has a relatively small slope, while for Δ>0.8, the slope is very sharp. In contrast, for 0≤Γ≤1, the change in parameter *K* is relatively uniform. In other words, parameter Γ does not change the character of the definition expression of parameter *K* (see (4)), while parameter Δ completely changes its character (see (6)).

Consequently, although some analytical results obtained using *K* and Δ can be corrected by replacing parameter Δ with Γ, using the relation $\Delta =2\Gamma /\left(1+\Gamma^{2}\right)$, parameterization based on a nonlinear relationship between $V_{1}$ and $V_{2}$ causes anomalies in graphical representations of PDF and ASEP expressions. Namely, corresponding ASEP curves are indistinguishably dense spaced for the entire range of Δ<0.5, which can be clearly observed from ([15], Figure 3) and ([2], Figure 7). In addition, the shapes of the corresponding PDF curves for all Δ<0.5 are almost the same as the shape of a Rician PDF curve obtained for Δ=0 and the same value of *K*, which is evident from ([1], Figure 7) and ([2], Figure 3). Therefore, its obvious that Δ-based parameterization does not clearly reflect the impact of the ration between $V_{1}$ and $V_{2}$ on the PDF shape and ASEP values.

Consequently, in most TWDP literature, PDF and ASEP curves are plotted only for specific values of Δ, i.e., Δ=0.5 and Δ=1, for which the mentioned differences can be easily distinguished, thus avoiding graphical presentation and explanation of the results for 0≤Δ≤0.5.

On the contrary, within the expressions obtained by integration or derivation with respect to parameter Δ (e.g., expression for Cramer–Rao bound of a moment-based estimator, etc.), simple replacement of Δ with Γ can not be performed, since different parameterizations completely change the behavior of involved expressions. In these situations, it is necessary to entirely reconsider existing TWDP and TWDP-related results.

Accordingly, considering conducted elaboration, TWDP fading in this paper will be characterized by parameters *K* and Γ.

### 2.2. Envelope PDF and CDF Expressions

To provide a mathematically convenient tool for TWDP performance evaluation, alternative exact envelope PDF and CDF expressions are proposed. Namely, it is noticed that assumptions about statistical characteristics of a complex envelope in a TWDP fading channel given in (1) are the same as those from [30,31] where the sum of signal, cochannel interference, and AWGN is modeled. However, unlike the existing approximate TWDP PDF and CDF expressions, PDF and CDF in [30,31] are given in the exact form. Accordingly, using ([30], Equation (6)) and ([31], Equation (12)) and considering adopted parameterization, we propose the following TWDP envelope PDF and CDF expressions:

(7) $$\begin{array}{cc} f_{R}\left(r\right) & =\frac{r}{\sigma^{2}}\exp\left(-\frac{r^{2}}{2\sigma^{2}}-K\right)\sum_{m=0}^{\infty }\varepsilon_{m}{\left(-1\right)}^{m}\\ & \times I_{m}\left(2r\sqrt{\frac{K}{2\sigma^{2}}\frac{1}{1+\Gamma^{2}}}\right)I_{m}\left(2r\sqrt{\frac{K}{2\sigma^{2}}\frac{\Gamma^{2}}{1+\Gamma^{2}}}\right)I_{m}\left(2K\frac{\Gamma }{1+\Gamma^{2}}\right) \end{array}$$

and

(8) $$\begin{array}{cc} F_{R}\left(r\right) & =\frac{r^{2}}{2\sigma^{2}}\exp\left(-\frac{r^{2}}{2\sigma^{2}}\right)\sum_{m=0}^{\infty }\frac{{\left(-1\right)}^{m}}{m!}{\left(\frac{K}{1+\Gamma^{2}}\right)}^{m}\\ & \times {}_{1}F_{1}\left(1-m;2;\frac{r^{2}}{2\sigma^{2}}\right){}_{2}F_{1}\left(-m,-m;1;\Gamma^{2}\right) \end{array}$$

where $0\leq V_{2}\leq V_{1}$, $\varepsilon_{0}=1$, $\varepsilon_{m}=2$ for m≥1, $I_{\nu }\left(\cdot \right)$ is a modified ν-th order Bessel function of the first kind, while ${}_{1}F_{1}\left(\cdot ;\cdot ;\cdot \right)$ and ${}_{2}F_{1}\left(\cdot ,\cdot ;\cdot ;\cdot \right)$ are confluent and Gaussian hypergeometric functions, respectively.

Note that the derived expressions ((7) and (8)) are given in terms of Bessel and hypergeometric functions, which can be easily evaluated and efficiently programmed in most standard software packages (e.g., Matlab, Maple and Mathematica) [18].

#### 2.2.1. Special Cases of a TWDP Model

It is easy to show that (7) and (8) can be reduced to Rayleigh and Rician PDF and CDF expressions.

The Rayleigh model assumes the absence of specular and the presence of only diffuse multipath components. It can be obtained from TWDP fading for $V_{1}=V_{2}=0$, i.e., K=0. Thus, by applying K=0 into (7) and (8), with $I_{\nu }\left(0\right)=0$ for ν≠0 and $I_{0}\left(0\right)=1$, (7) and (8) can be reduced to Rayleigh PDF and CDF expressions:

(9) $$f_{R}\left(r\right){|}_{K=0}=\frac{r}{\sigma^{2}}\exp\left(-\frac{r^{2}}{2\sigma^{2}}\right)$$

(10) $$F_{R}\left(r\right){|}_{K=0}=\frac{r^{2}}{2\sigma^{2}}\exp\left(-\frac{r^{2}}{2\sigma^{2}}\right)$$

respectively.

Rician fading assumes the presence of one specular component and many diffuse components. It can be obtained from TWDP fading for $V_{2}=0$, i.e., Γ=0. In this case, (7) can be reduced to a well-known Rician PDF expression:

(11) $$f_{R}\left(r\right){|}_{\Gamma =0}=\frac{r}{\sigma^{2}}\exp\left(-\frac{r^{2}}{2\sigma^{2}}-K\right)I_{0}\left(2r\sqrt{\frac{K}{2\sigma^{2}}}\right)$$

Additionally, by inserting Γ=0 into (8) and considering that ${}_{2}F_{1}\left(\cdot ,\cdot ;\cdot ;0\right)=1$ and ${}_{1}F_{1}\left(1;2;x\right)=\left(e^{x}-1\right)/x$, TWDP CDF reduces to:

(12) $$\begin{array}{cc} F_{R}\left(r\right){|}_{\Gamma =0} & =1-\exp\left(-\frac{r^{2}}{2\sigma^{2}}\right)+\frac{r^{2}}{2\sigma^{2}}\exp\left(-\frac{r^{2}}{2\sigma^{2}}\right)\\ & \times \sum_{m=1}^{\infty }\frac{{\left(-1\right)}^{m}}{m!}K^{m}{}_{1}F_{1}\left(1-m;2;\frac{r^{2}}{2\sigma^{2}}\right) \end{array}$$

which, according to ([33], Equation (8.352.1)), ([33], Equation (8.972.1)) and ([34], Equation (12)), takes the well-known form of a Rician CDF, expressed in terms of the first-order Marcum Q-function $Q_{1}\left(\cdot ,\cdot \right)$ [2]:

(13) $$F_{R}\left(r\right){|}_{\Gamma =0}=1-Q_{1}\left(\sqrt{2K},\frac{r}{\sigma }\right),$$

#### 2.2.2. Convergence Analysis

It is also easy to show that (7) and (8), as infinite-series expressions, are convergent.

To prove convergence of (7), the d’Alembert’s ratio test is used. According to the test, the infinite-series $\sum_{k}c_{k}$ is convergent if the limiting expression $\lim_{k\to \infty }\left|c_{k+1}/c_{k}\right|$ is smaller than 1. Thus, the ratio test applied to (7) yields the following expression:

(14) $$\begin{array}{c} \lim_{k\to \infty }|\frac{c_{k+1}}{c_{k}}|=\lim_{k\to \infty }[\frac{I_{k+1}\left(2r\sqrt{\frac{K}{2\sigma^{2}}\frac{1}{1+\Gamma^{2}}}\right)}{I_{k}\left(2r\sqrt{\frac{K}{2\sigma^{2}}\frac{1}{1+\Gamma^{2}}}\right)}\\ \times \frac{I_{k+1}\left(2r\sqrt{\frac{K}{2\sigma^{2}}\frac{\Gamma^{2}}{1+\Gamma^{2}}}\right)I_{k+1}\left(2K\frac{\Gamma }{1+\Gamma^{2}}\right)}{I_{k}\left(2r\sqrt{\frac{K}{2\sigma^{2}}\frac{\Gamma^{2}}{1+\Gamma^{2}}}\right)I_{k}\left(2K\frac{\Gamma }{1+\Gamma^{2}}\right)}] \end{array}$$

which can be calculated using ([35], Equation (3.12)) as:

(15) $$\begin{array}{c} \lim_{k\to \infty }|\frac{c_{k+1}}{c_{k}}|=\lim_{k\to \infty }[\frac{\left(2r\sqrt{\frac{K}{2\sigma^{2}}\frac{1}{1+\Gamma^{2}}}\right)}{\left(2r\sqrt{\frac{K}{2\sigma^{2}}\frac{1}{1+\Gamma^{2}}}+k\right)}\\ \times \frac{\left(2r\sqrt{\frac{K}{2\sigma^{2}}\frac{\Gamma^{2}}{1+\Gamma^{2}}}\right)}{\left(2r\sqrt{\frac{K}{2\sigma^{2}}\frac{\Gamma^{2}}{1+\Gamma^{2}}}+k\right)}\frac{\left(2K\frac{\Gamma }{1+\Gamma^{2}}\right)}{\left(2K\frac{\Gamma }{1+\Gamma^{2}}+k\right)}]=0<1 \end{array}$$

The above expression shows that the series in (7) is convergent.

Similarly, the convergence of CDF (8) is also proven using d’Alambert’s ratio test, with its kth term denoted by $c_{k}$. Since ${}_{2}F_{1}\left(-k,-k;1;\Gamma^{2}\right)$ is kth order polynomial, due to ([33], Equation (8.822-4), (8.911-1), (8.917.1)), it can be written as $\left(\left(2k\right)!{\left(1+\Gamma^{2}\right)}^{k}\right)/\left(2^{k}{\left(k!\right)}^{2}\right)+O\left(x^{k-1}\right)$. Furthermore, following ([33], Equation (8.970-1), (8.972-1)), it is evident that ${}_{1}F_{1}\left(1-k;2;\frac{r^{2}}{2\sigma^{2}}\right)$ is also a kth order polynomial dominated by 1/k when $r^{2}\leq 2\sigma^{2}$ and by $[{\left(\left(-r^{2}\right)/\left(2\sigma^{2}\right)\right)}^{\left(k-1\right)}]/k!$ when $r^{2}>2\sigma^{2}$. Considering the above, d’Alambert’s ratio test yields:

$$\lim_{k\to \infty }|\frac{c_{k+1}}{c_{k}}|=\left\{\begin{array}{cc} \lim_{k\to \infty }\left(K\frac{r^{2}}{2\sigma^{2}}\frac{\left(2k+1\right)}{{\left(k+1\right)}^{3}}\right), & r^{2}>2\sigma^{2}\\ \lim_{k\to \infty }\left(K\frac{(2k+1)k}{{\left(k+1\right)}^{3}}\right), & r^{2}\leq 2\sigma^{2} \end{array}\right.$$

which is always equal to zero and thus smaller than one. Therefore, the series in (8) is also convergent.

#### 2.2.3. Graphical Results

In order to investigate the accuracy of (7) and (8) and their applicability for modeling various fading conditions, Equations (7) and (8) are plotted for different sets of TWDP parameters.

**Figure 4:**
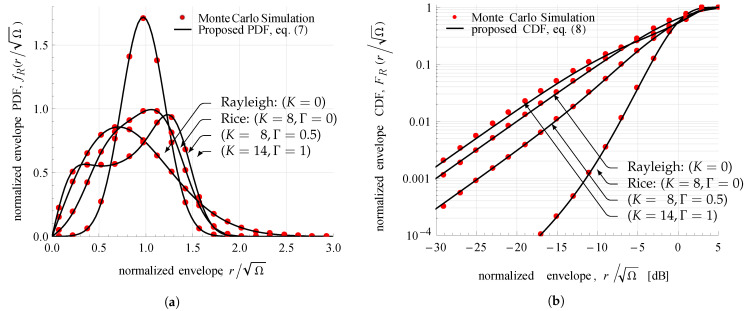
TWDP normalized envelope (**a**) PDF and (**b**) CDF curves for various combinations of *K* and Γ.

Equation (7) is used to plot the normalized envelope PDF, $f_{R}\left(r/\sqrt{\Omega }\right)$, for different fading conditions: Rician with K=8 and Γ=0; Rayleigh with K=0; and others, with K=8 and Γ=0.5; and K=14 and Γ=1. Figure 4a depicts these curves together with corresponding normalized histograms created by Monte Carlo simulation. All curves are obtained by limiting truncation error below $10^{-6}$, i.e., by employing up to 35 summation terms in all tested cases. Each normalized histogram, composed of 20 equally spaced bins, is computed independently by generating $10^{6}$ samples for the considered fading conditions. Figure 4a shows matching results between the analytical and simulated approaches, thus validating the proposed PDF expression in diverse fading conditions.

Figure 4b compares normalized envelope CDF curves $F_{R}\left(r/\sqrt{\Omega }\right)$ obtained from (8) with normalized cumulative histograms. Similarly, Monte Carlo simulation is used to generate histograms with the same set of parameters as in the PDF comparison. Analytically obtained curves are generated by employing up to 118 summation terms in order to achieve a truncation error of less than $10^{-26}$. Normalized cumulative histograms are created from $10^{6}$ samples divided into 20 bins. The conducted comparison shows matching results between the analytical and simulated approaches, thus demonstrating the applicability of (8) for accurate calculation of CDF values in different fading conditions.

## 3. Alternative form of TWDP SNR MGF Expression

In this section, the alternative form of the MGF of the SNR is derived based on the proposed CDF expression. Here, the well-known relationship between CDF and MGF is used ([36], Equation (1.2)):

(16) $$\begin{array}{cc} M_{\gamma }\left(s\right) & =\int_{0}^{+\infty }f_{\gamma }\left(\gamma \right)\exp\left(s\gamma \right)d\gamma \\ & =L\left\{f_{\gamma }\left(\gamma \right);\gamma ,-s\right\}\\ & =L\left\{\frac{d}{d\gamma }F_{\gamma }\left(\gamma \right);\gamma ,-s\right\}\\ & =-sL\left\{F_{\gamma }\left(\gamma \right);\gamma ,-s\right\}-F_{\gamma }\left(\gamma =0\right)\\ & =-sL\left\{F_{\gamma }\left(\gamma \right);\gamma ,-s\right\} \end{array}$$

where $L\left\{h\left(t\right);t,p\right\}≜\int_{0}^{\infty }h\left(t\right)e^{-pt}dt$ represents Laplace transform of h(t) from the *t*-domain into the *p*-domain, and $F_{\gamma }\left(\gamma \right)$ is the CDF of the SNR. $F_{\gamma }\left(\gamma \right)$ is obtained from (8) according to the random variable transformation $\gamma =r^{2}\frac{E_{s}}{N_{0}}$, as:

(17) $$\begin{array}{cc} F_{\gamma }\left(\gamma \right) & =\frac{\gamma }{\gamma_{0}}\left(1+K\right)\exp\left(-\frac{\gamma }{\gamma_{0}}\left(1+K\right)\right)\sum_{m=0}^{\infty }\frac{{\left(-1\right)}^{m}}{m!}\\ & \times {\left(\frac{K}{1+\Gamma^{2}}\right)}^{m}{}_{1}F_{1}\left(1-m;2;\frac{\gamma }{\gamma_{0}}\left(1+K\right)\right){}_{2}F_{1}\left(-m,-m;1;\Gamma^{2}\right) \end{array}$$

where $\gamma_{0}=2\sigma^{2}\left(1+K\right)\frac{E_{s}}{N_{0}}$ is the average SNR, $E_{s}$ denotes symbol energy, and $N_{0}/2$ is the power spectral density of the white Gaussian noise.

For simplicity, (17) is expressed in the following form:

(18) $$\begin{array}{c} F_{\gamma }\left(\gamma \right)=\sum_{m=0}^{\infty }A\gamma B_{m}\exp\left(-A\gamma \right){}_{1}F_{1}\left(1-m;2;A\gamma \right) \end{array}$$

where $B_{m}={\left(-K/(1+\Gamma^{2})\right)}^{m}{}_{2}F_{1}\left(-m,-m;1;\Gamma^{2}\right)/m!$ and $A=\left(1+K\right)/\gamma_{0}$. Based on ([37], Equation (07.20.16.0001.01)), (18) is further simplified as:

(19) $$F_{\gamma }\left(\gamma \right)=\sum_{m=0}^{\infty }A\gamma B_{m}{}_{1}F_{1}\left(1+m;2;-A\gamma \right)$$

Laplace transform of (19) is then obtained using ([38], Equation (3.35.1-2)) as:

(20) $$L\left\{F_{\gamma }\left(\gamma \right);\gamma ,s\right\}=\sum_{m=0}^{\infty }\frac{AB_{m}}{s^{2}}{}_{2}F_{1}\left(1+m;2;2,-\frac{A}{s}\right)$$

which, according to ([37], Equation (07.23.03.0080.01)), can be expressed in the following form:

(21) $$\begin{array}{c} L\left\{F_{\gamma }\left(\gamma \right);\gamma ,s\right\}=\sum_{m=0}^{\infty }\frac{AB_{m}}{{\left(A+s\right)}^{2}}{\left(\frac{s}{A+s}\right)}^{m-1} \end{array}$$

Finally, by combining (16) and (21), the MGF is derived as:

(22) $$\begin{array}{cc} M_{\gamma }\left(s\right) & =\frac{1+K}{1+K-s\gamma_{0}}\sum_{m=0}^{\infty }\frac{1}{m!}{\left(\frac{K}{1+\Gamma^{2}}\right)}^{m}{\left(\frac{\gamma_{0}s}{1+K-s\gamma_{0}}\right)}^{m}{}_{2}F_{1}\left(-m,-m;1;\Gamma^{2}\right) \end{array}$$

which represents an alternative form of the exact TWDP MGF of the SNR.

It can be proven that (22) can be easily transformed into the well-known TWDP MGF expression form ([16], Equation (25)) (originally given in terms of *K* and Δ). Namely, by using the identity between the Gaussian hypergeometric function and the Legendre polynomial given by ([39], Equation (15.4.14)), as well as the identity between the Legendre polynomial and the first-kind zero-order Bessel function given by ([40], Equation (0.6)), and after some simple manipulations, it can be shown that:

(23) $$\sum_{m=0}^{\infty }\frac{1}{m!}a^{m}{}_{2}F_{1}\left(-m,-m;1;b\right)=\exp\left(a+ab\right)I_{0}\left(2a\sqrt{b}\right)$$

Therefore, by using (23), (22) can be written as:

(24) $$\begin{array}{cc} M_{\gamma }\left(s\right) & =\frac{1+K}{1+K-\gamma_{0}s}\exp\left(\frac{\gamma_{0}Ks}{1+K-\gamma_{0}s}\right)I_{0}\left(\frac{2\Gamma }{1+\Gamma^{2}}\frac{\gamma_{0}Ks}{1+K-\gamma_{0}s}\right) \end{array}$$

which is the same expression as the verified SNR MGF from [16], only expressed in terms of *K* and Γ.

Although simple, the analytical form of MGF expressed by (24) has not been often used for error rate performance evaluation in TWDP fading channels. The main disadvantage with this expression is its unfavorable analytical form for mathematical manipulations. In contrast, the analytical form of MGF as expressed by (22) enables derivation of the exact expressions for the performance evaluation in a variety of TWDP fading conditions.

## 4. Error Probability of M-ary PSK Receiver in TWDP Fading Channel

### 4.1. The Exact M-ary PSK ASEP Expression

This section demonstrates the applicability of the proposed TWDP SNR MGF (22) for derivation of the exact M-ary PSK ASEP expression, where M represents the order of PSK modulation.

M-ary PSK ASEP in a TWDP fading channel can be determined from ([36], Equation (5.78)):

(25) $$P_{s}\left(\gamma_{0}\right)=\frac{1}{\pi }\int_{0}^{\pi -\frac{\pi }{M}}M_{\gamma }\left(-\frac{\sin^{2}\frac{\pi }{M}}{\sin^{2}\theta }\right)d\theta$$

where $M_{\gamma }\left(\cdot \right)$ represents the MGF of the SNR given in (22). Accordingly, Equation (25) can be expressed as $P_{s}\left(\gamma_{0}\right)=2I{{|}_{0}^{\frac{\pi }{2}}-I|}_{0}^{\frac{\pi }{M}}$, where I represents the indefinite integral defined as:

(26) $$\begin{array}{cc} I & =\frac{1}{\pi }\int M_{\gamma }\left(-\frac{\sin^{2}\frac{\pi }{M}}{\sin^{2}\theta }\right)d\theta \\ & =\frac{1}{\pi }\sum_{m=0}^{\infty }\frac{1}{m!}{\left(\frac{K}{1+\Gamma^{2}}\right)}^{m}{}_{2}F_{1}\left(-m,-m;1;\Gamma^{2}\right)\\ & \times \int \left(\frac{1+K}{1+K-\gamma_{0}s}\right){\left(\frac{\gamma_{0}s}{1+K-\gamma_{0}s}\right)}^{m}d\theta {|}_{s=-\frac{\sin^{2}\frac{\pi }{M}}{\sin^{2}\theta }} \end{array}$$

which can be solved using Wolfram Mathematica as:

(27) $$\begin{array}{cc} I & =\frac{1}{3\pi }\frac{1+K}{\gamma_{0}}\sum_{m=0}^{\infty }\frac{{\left(-1\right)}^{m}}{m!}{\left(\frac{K}{1+\Gamma^{2}}\right)}^{m}\frac{\sin^{3}\theta }{\sin^{2}\pi /M}\\ & \times {AF}_{1}\left(\frac{3}{2};\frac{1}{2},1+m;\frac{5}{2};\sin^{2}\theta ,-\frac{1+K}{\gamma_{0}}\frac{\sin^{2}\theta }{\sin^{2}\frac{\pi }{M}}\right)\\ & \times {}_{2}F_{1}\left(-m,-m;1;\Gamma^{2}\right),\mathrm{for}0\leq \theta \leq \frac{\pi }{2} \end{array}$$

where $AF_{1}\left(\cdot ;\cdot ,\cdot ;\cdot ;\cdot ,\cdot \right)$ is an Appell hypergeometric function. Considering the above, the integral in (25) can be solved as:

(28) $$\begin{array}{cc} P_{s} & \left(\gamma_{0}\right)=\frac{\sin\frac{\pi }{M}}{3\pi }\frac{1+K}{\gamma_{0}}\sum_{m=0}^{\infty }\frac{1}{m!}{}_{2}F_{1}\left(-m,-m;1;\Gamma^{2}\right)\\ & \times {\left(\frac{-K}{1+\Gamma^{2}}\right)}^{m}\left[\frac{3\pi }{2\sin^{3}\frac{\pi }{M}}{}_{2}F_{1}\left(\frac{3}{2},1+m;2;-\frac{1+K}{\gamma_{0}\sin\frac{\pi }{M}}\right)\right.\\ & \left.-AF_{1}\left(\frac{3}{2};\frac{1}{2},1+m;\frac{5}{2};\sin^{2}\frac{\pi }{M},-\frac{1+K}{\gamma_{0}}\right)\right] \end{array}$$

which represents M-ary PSK ASEP given as the exact analytical expression.

Similarly as in the cases of PDF and CDF expressions, derived M-ary PSK ASEP (28) is also given in terms of standard mathematical functions, which can be easily evaluated and efficiently programmed in standard software packages [18].

### 4.2. Asymptotic Expression of M-ary PSK ASEP

To gain further insight into the TWDP M-ary PSK ASEP behavior, the asymptotic ASEP for large values of $\gamma_{0}$ is derived. Furthermore, this allows us to relax the computational complexity which occurs for large values of *K*.

Considering that $AF_{1}\left(a;b_{1},b_{2};c;z_{1},z\right)∼{}_{2}F_{1}\left(a,b_{1};c;z_{1}\right)$ and ${}_{2}F_{1}\left(a,b;c;z\right)∼1$ when $z\overset{}{\to }0$, Equation (28) for large values of $\gamma_{0}$ can be expressed as:

(29) $$\begin{array}{cc} P_{s}\left(\gamma_{0}\right) & \approx \frac{\sin\frac{\pi }{M}}{3\pi }\frac{1+K}{\gamma_{0}}\sum_{m=0}^{\infty }\frac{{\left(\frac{-K}{1+\Gamma^{2}}\right)}^{m}}{m!}{}_{2}F_{1}\left(-m,-m;1;\Gamma^{2}\right)\\ & \times \left[\frac{3\pi }{2\sin^{3}\frac{\pi }{M}}-{}_{2}F_{1}\left(\frac{3}{2},\frac{1}{2};\frac{5}{2};\sin^{2}\frac{\pi }{M}\right)\right] \end{array}$$

Equation (29) can be further simplified using the identity ([41], p. 24) and Equation (23), as:

(30) $$\begin{array}{cc} P_{s}\left(\gamma_{0}\right)\approx & \frac{1+K}{2\pi \gamma_{0}}\frac{\pi -\frac{\pi }{M}+\frac{1}{2}\sin\frac{2\pi }{M}}{\sin^{2}\frac{\pi }{M}}e^{-K}I_{0}\left(\frac{2\Gamma K}{1+\Gamma^{2}}\right) \end{array}$$

which represents a simple, closed-form asymptotic M-ary PSK ASEP expression. By inserting Γ=0 and K=0 into (30), the asymptotic expression for M-ary PSK ASEP in the TWDP fading channel takes the well-known form of asymptotic ASEP expressions in Rician ([42], Equation (62)) and Rayleigh ([43], Equation (9)) fading channels, respectively:

(31) $$\begin{array}{cc} P_{s}\left(\gamma_{0}\right){|}_{\Gamma =0}\approx & \frac{1+K}{2\pi \gamma_{0}}\frac{\pi -\frac{\pi }{M}+\frac{1}{2}\sin\frac{2\pi }{M}}{\sin^{2}\frac{\pi }{M}}e^{-K} \end{array}$$

(32) $$\begin{array}{cc} P_{s}\left(\gamma_{0}\right){|}_{K=0}\approx & \frac{1}{2\pi \gamma_{0}}\frac{\pi -\frac{\pi }{M}+\frac{1}{2}\sin\frac{2\pi }{M}}{\sin^{2}\frac{\pi }{M}} \end{array}$$

### 4.3. Numerical Results

In order to validate the conducted error performance analysis and to justify the proposed parameterization, this section provides a graphical interpretation of analytically derived M-ary PSK ASEP and its comparison to results obtained by Monte Carlo simulation. Different modulation orders and TWDP parameters are investigated.

**Figure 5:**
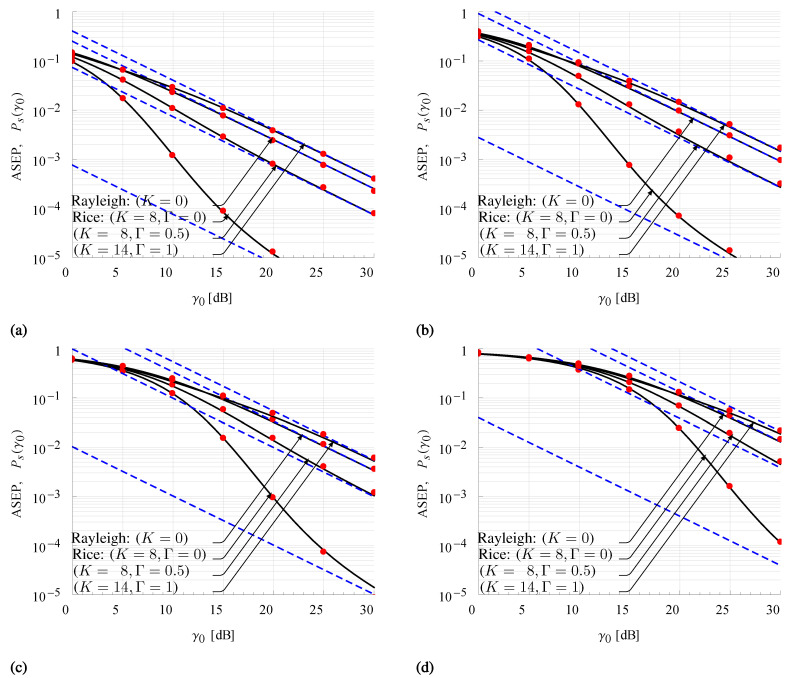
Exact (solid line) and asymptotic (dashed line) expressions of TWDP ASEP for (**a**) 2-PSK, (**b**) 4-PSK, (**c**) 8-PSK and (**d**) 16-PSK modulations compared with Monte Carlo simulation results (dots).

Figure 5a–d illustrate the exact (28) and the asymptotic (30) ASEP for 2-PSK, 4-PSK, 8-PSK, and 16-PSK modulations for a set of previously adopted TWDP parameters. ASEP curves, obtained from (28) by limiting truncation error to $10^{-6}$, i.e., by employing up to 78 summation terms, are compared with those obtained using Monte Carlo simulations generated with $10^{6}$ samples. Matching results between the exact and simulated ASEP, as well as between the exact and high-SNR asymptotic ASEP, can be observed for the considered modulation orders and the set of TWDP parameters. Accordingly, derived ASEP expressions can be used to accurately evaluate the error probability of the M-ary PSK receiver for all fading conditions implied by the TWDP model.

Based on the above, a comparison of error performance of channels with different fading severities is also performed following Figure 5a–d. Clearly, the signal in the fading condition characterized with K=14, Γ=1 exhibits worse performance compared to the Rayleigh fading channel (K=0), thus representing a signal in near hyper-Rayleigh fading conditions. It also can be observed that ASEP in fading conditions described with the same value of *K* increases with increasing Γ, indicating that signal performance significantly degrades in channels with Γ=0.5 with respect to those in typical Rician channels (Γ=0).

**Figure 6:**
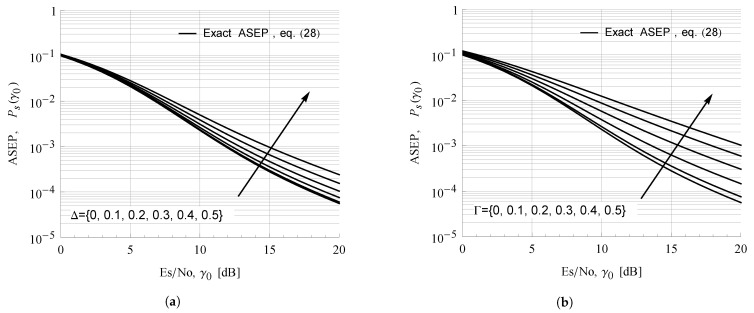
BPSK ASEP in TWDP channel for K=6 and different values of parameter (**a**) Δ (**b**) Γ.

Figure 6 illustrates the effect of proposed parameterization on 2-PSK ASEP curves in the TWDP fading channel with K=6. Obviously, Γ-based parameterization solved the problem of densely-spaced ASEP curves observed for the entire range of Δ between 0 and 0.5, enabling us to clearly and unequivocally observe the impact of the ratio between specular components on ASEP values.

## 5. Conclusions

This paper proposed a novel analytical characterization of TWDP fading channels achieved by introducing physically justified TWDP parameterization and exact PDF and CDF expressions, and by deriving the alternative form of the exact SNR MGF expression. Benefits of the proposed parameterization are demonstrated on TWDP PDF and ASEP graphical interpretations. A derived MGF is used for derivation of the exact M-ary PSK ASEP expression, which can be used to accurately evaluate the error performance of M-ary PSK in various fading conditions.

## Data Availability

Not applicable.
